# Variants of the *CASP9* gene as candidate markers for primary response to anti-TNF therapy in Crohn’s disease patients

**DOI:** 10.1007/s13353-023-00783-7

**Published:** 2023-09-02

**Authors:** Liliana Lykowska-Szuber, Michal Walczak, Kamila Stawczyk-Eder, Iwona Krela-Kazmierczak, Piotr Eder, Oliwia Zakerska-Banaszak, Agnieszka Dobrowolska, Marzena Skrzypczak-Zielinska

**Affiliations:** 1 Department of Gastroenterology, Dietetics and Internal Diseases, Poznan University of Medical Sciences, Przybyszewskiego 49, 60-355 Poznan, Poland; 2 Institute of Human Genetics, Polish Academy of Sciences, Poznan, Poland

**Keywords:** Crohn’s disease, Biological therapy, *CASP9* gene, Anti-TNF mAbs, Response

## Abstract

Anti-tumor necrosis factor (TNF) therapy is used to induce and maintain remission in Crohn’s disease (CD) patients. However, primary non-responders to initial treatment constitute 20–40% of cases. The causes of this phenomenon are still unknown. We aim to investigate the impact of the caspase 9 (*CASP9*) gene variants on the variable reactions of CD patients to anti-TNF therapy. The study group included 196 diagnosed and clinically characterized CD Polish patients following anti-TNF therapy. The sequence of the *CASP9* gene was analyzed using next-generation and Sanger sequencing and was analyzed with the response to biological treatment. Using the RT-qPCR analysis, we estimated the *CASP9* gene mRNA level in colon biopsies material from inflamed and non-inflamed tissue (21 CD patients: 14 responders and seven non-responders to anti-TNF therapy and six controls), as well as in vitro in a peripheral blood mononuclear cells (PBMCs) from CD patients (seven responders and seven non-responders to anti-TNF therapy) and eight controls. Our findings indicated association of variants rs1052571 and rs4645978 with response to anti-TNF monoclonal antibodies (mAbs). Moreover, we observed tendency for reduced expression after incubation with anti-TNF in the group of CD patients, in contrast to the control group. Our results suggest that response to anti-TNF therapy in CD patients may be an effect of variants of the *CASP9* gene as a key effector of the internal pathway of apoptosis; however, further population and functional research are necessary.

**Supplementary Information:**

The online version contains supplementary material available at 10.1007/s13353-023-00783-7.

## Introduction

With the development of societies industrialization, we observe an increase in the incidence of inflammatory bowel diseases (IBD). The onset of the disease usually falls around the age of 20, i.e., the period of the greatest life activity, which is why the challenge of modern medicine is to implement effective treatment returning patients to full agility. Crohn’s disease (CD) is a disorder that can lead to permanent disability. It has been proven that properly selected and intensive therapy, introduced at the earliest possible stage of the disease, can prevent its unfavorable development. Biological drugs, including anti-TNF mAbs, have become a hope for effective treatment. The Food and Drug Administration approved them for CD in 1998 which revolutionized the IBD treatment.

The cytokine TNFα is one of the primary cytokines responsible for activating and maintaining inflammation in the altered gastrointestinal tract tissues in patients with CD (Van Assche and Rutgeerts 2000). Increased secretion of it was found in the inflamed mucosa, the lamina propria, and the submucosa (Murch et al. 1993). TNF is involved, among others, in the accumulation of neutrophils, the formation of granulomas as well as increased permeability of the intestinal epithelium (Mullin and Snock 1990; Amiri et al. 1992). Anti-TNF drugs bind to soluble and transmembrane TNFα and exhibit a particular affinity for inflamed tissues. The TNF transmembrane form is attached to the target cell surface and anti-TNF binds to it, acting as a ligand through reverse signaling, thereby suppressing cytokines and activating apoptosis. Anti-TNF drugs also induce apoptosis of activated T lymphocytes in the lamina propria. On this way, they inhibit one of the key disability responsible for the chronicity of inflammation in CD, increased survival and resistance to apoptosis of T lymphocytes (Van den Brande et al. 2007; Itoh et al. 2001). Studies up to date showed an imbalance in the controlled death of peripheral blood lymphocytes in both CD and ulcerative colitis (UC) (Dudzinska et al. 2019). Additionally, it was shown that apoptosis is the main mechanism by which infliximab exerts a killing activity on lamina propria T cells (LPT) in CD (Di Sabatino et al. 2004). Although anti-TNF drugs have changed the approach to treating patients with CD, it is still challenging to predict an individual patient’s response. Approximately 30% of patients do not respond to this form of therapy—“primary non-response” and nearly 50% will lose this effectiveness within the first year of therapy—“secondary non-response.” While the production of anti-drug antibodies is primarily responsible for the loss of the secondary response, the mechanisms responsible for the lack of the primary response are still not fully understood. Due to the development of new biological molecules, in recent years, there is an increasing need to develop biomarkers that will allow the initiation of personalized medicine and, as a result, will lead to a greater effectiveness of therapy, reducing risk of complications, and costs (Lykowska-Szuber et al. 2023a, b).

Therefore, the aim of our study was to investigate whether variants of caspase 9 (*CASP9)* gene, one of the key proteinases responsible for the induction of the internal pathway of cell apoptosis, may be one of the reasons for the lack of primary response to anti-TNF alpha therapy in patients with CD. Our goal was to answer this question by conducting both studies of the *CASP9* gene sequence and the tissue-specific CASP9 expression profile in the intestinal mucosa of CD patients treated with anti-TNF and in vitro studies in peripheral blood mononuclear cells (PBMCs) from CD patients cultured with anti-TNF mAbs. The current investigation in part of the *CASP9* gene sequence analysis is complementary (includes the same set of the studied DNA samples) to the previously published research estimating selected variants within *FCGR3A*, *IL1R*, *TNFSF1B*, *IL1B*, *FAS*, and *ADAM17* genes (Lykowska-Szuber et al. 2023a) and in part of *CASP9* gene expression is complementary (includes the same set of cDNA material) to previously described research on *FCGR3A*, *IL1R*, *TNFSF1B*, *IL1B*, *FAS*, and *ADAM17* genes expression genes (Lykowska-Szuber et al. 2021).

## Material and methods

### Clinical characterization and study

**Fig. 1:**
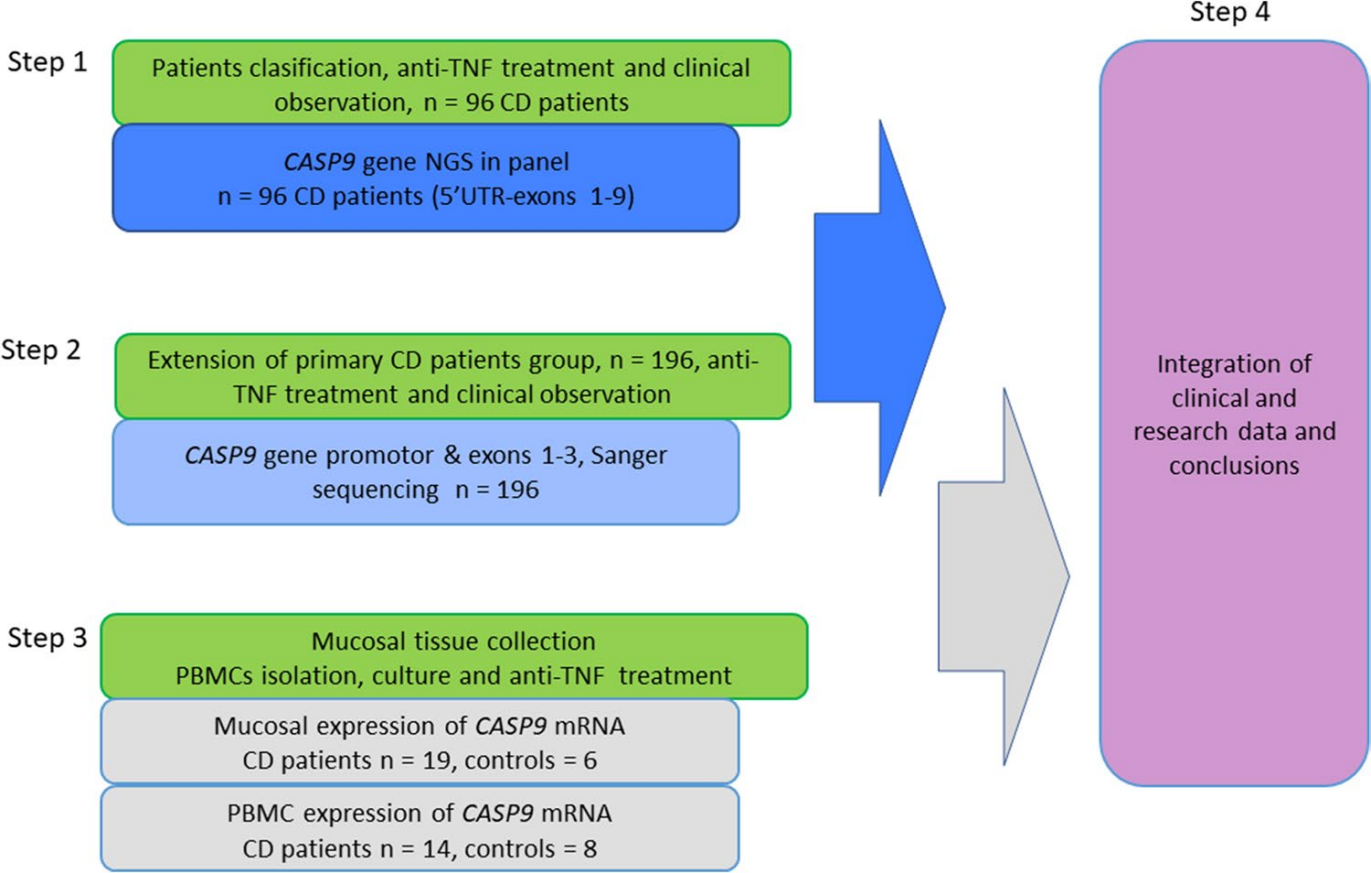
Flow chart of conducted research. UTR—untranslated region

The study included in total 196 Polish patients hospitalized at the Department of Gastroenterology, Dietetics and Internal Diseases of the Medical University in Poznan with a confirmed diagnosis of CD based on a history, physical examination, endoscopy, and MR enterography. All subjects were treated with anti-TNF therapy under the therapeutic program of the National Health Fund (the official reimbursement program for all biological therapies in Poland) at the Gastroenterology Clinic in years 2017–2021. We included in the study biologically naïve patients > 18 years old with active CD and after treatment failure or intolerance to first-line therapies such as mesalamine, corticosteroids and/or immunosuppressants. The exclusion criteria were the presence of an ileostomy or colostomy and infectious complications (including intraabdominal infections). The diagnosis was based on predefined criteria (Gomollón et al. 2017) and clinical disease activity was assessed using the Crohn's Disease Activity Index (CDAI) (Best et al. 1976). Individuals who had never smoked or quit smoking for at least 10 years prior to participating in the study were considered non-smokers. Patients were administered with infliximab (IFX) infusions at a dose of 5 mg/kg body weight at weeks 0, 2, 6 (induction phase) and then every 8 weeks up to a year (54 weeks—maintenance phase). Adalimumab (ADA) was administered subcutaneously at week 0 at a dose of 160 mg, 80 mg at week 2, then 40 mg every other week for up to 1 year (54 weeks). Response to anti-TNF treatment was assessed after 12 weeks of treatment. The CDAI score was used to determine the clinical response. Clinical response was defined as a reduction in CDAI of ≥ 70 points. In patients with fistulas, a complete response was defined as complete cessation of drainage of all fistulas, while a partial response was defined as a reduction of at least 50%, but not drainage of all fistulas. We also assessed the biological parameter (C-reactive protein, CRP), endoscopic response (simple endoscopic assessment of Crohn’s disease, SES-CD) and MRI (simple assessment of enterographic activity in Crohn’s disease, SEAS-CD) (Daperno et al. 2004; Eder et al. 2013a). These parameters were assessed twice—before treatment and after 12 weeks of induction therapy. The scheme of our study consisted of four stages and it is shown in Fig. 1.

In the first step, after DNA isolation from collected peripheral blood samples, we performed sequencing of the *CASP9* gene (from 5′UTR to exon 9 with 3′ untranslated region, UTR) in group of 96 CD patients (86 responders and 10 non-responders to anti-TNF mAbs) using NGS technology. In the second step of our research after NGS results interpretation and enlargement of CD patients group to 196 subjects treated with anti-TNF, we performed analysis of the *CASP9* gene promotor with exons 1, 2 and 3. Table S1 in supplementary material presents whole patients group characteristics. The third step of current research involving *CASP9* gene expression in CD patients in two types of material was carried out. The first group consisted of 19 individuals, for whom the research was carried out on tissue material collected during a routine colonoscopy. The second group included 13 patients, from whom peripheral blood samples for cell culture studies were collected. The detailed clinical characteristics of both studied groups are presented in supplementary material (Table S2 and Table S3). The control groups for *CASP9* gene expression studies consisted of 6 healthy (2 female, 4 male) subjects with the average age of 46.7 years in mucosal studies and 8 healthy subjects (4 female, 4 male) with average age 40.2 years in PBMCs culture studies.

All subjects gave their written consent to carry out genetic testing, endoscopy, MR enterography, and serum testing for the assessment of biochemical parameters. The research was approved by the Bioethics Committee of the Medical University in Poznan under Resolution No. 762/13 approved on 9 November 2013 and Resolution No. 1042/18 approved on 11 October 2018. All experiments were performed in accordance with the principles of the 1964 Declaration of Helsinki with its later amendments.

### DNA isolation, next generation, and Sanger sequencing analysis

Genomic DNA was isolated from the peripheral blood of all participants using the method with guanidine isothiocyanate and stored at 4 °C in a TE buffer containing 1 mM EDTA and 10 mM Tris–Cl. Next, the amplification of *CASP9* gene regions were performed using PCR primers sequences presented in Table 1.

**Table 1:** Primers details *CASP9* gene

*CASP9* region	Method	Coordinates (GRCh38/ hg38)	Sequences 5′ → 3′	Product lenght bp	Anne. temp. °C
5′ flanking—exon 3*	NGS	chr1:15517640-15527223	F: CAGCATATCCAGGACCAGGAAGAAG R: GGTCTAATGATACCTGCCTTGCAAGG	9584	65
Exons 4–7*	NGS	chr1:15498561-15508228	F: AACTGTTGTTAGATTGGTTGTTTCCA R: AACTGTCCACTATATCATAGGCACTC	9668	65
Exons 8–9*	NGS	chr1:15492057-15499341	F:CTGCAGCTTTCTACAGTTCTCTAATG R:AATAAGACATGAATAAATGGGGCTGG	7285	65
Promoter Part 1	Sanger	chr1:15525230-15525613	F: GAACGATGGGAGTTTACAGGAC R: CAATTCTGGCACCAGTTCCT	384	63
Promoter Part 2	Sanger	chr1:15524787-15525252	F: ACCAGGAACTGGTGCCAGAA R: ATCTAGAAGGTCTCGCCCCG	466	63
Promoter Part 3	Sanger	chr1:15524400-15524806	F: CGGGGCGAGACCTTCTAGATGC R: CGCCCTCAGGACGCACCTCT	407	55
Exon 1	Sanger	chr1:15523925-15524337	F: GGTGGGGAGCGAAGACTGACC R: ACACCCGACTAAGAGGTGTTTG	413	64
Exon 2	Sanger	chr1:15517919-15518551	F: GCAAAGGCTGGTAAATGGCA R: ATGCTTTTCAGAGGAGGGGC	633	60
Exon 3	Sanger	chr1:15523989-15524336	F: GTGGGGAGCGAAGACTGAC R: GCTAGGCTCCCGCACAAC	348	60

^*^Primers and PCR conditions previously described in Walczak et al 2019; *F*, primer forward; *R*, primer reverse; *Anne. temp.*, annealing temperature

For NGS amplicon library preparation based on a total of 3 amplicons from each patient were prepared using previously described conditions (Walczak et al. 2019). According to the manufacturer’s protocol, 1 ng of the pooled DNA fragments was subjected to the Nextera XT procedure (Illumina) using transposome technology. Finally, using the Nextera XT DNA Sample Preparation Kit (Illumina) and the Nextera® XT Index Kit (96) (Illumina), we obtained one hundred and seven both-side indexed DNA libraries ready for high-throughput sequencing. The normalization of all libraries was carried out with magnetic beads, according to the Nextera XT procedure. Sequencing on the Illumina MiSeq platform was performed as paired-end targeted resequencing using the MiSeq Reagent Kit v2 (300 cycle) (Illumina).

PCR program for amplification of the promotor regions and exons 1, 2, and 3 for Sanger sequencing, started with initial denaturation at 95 °C for 4 min, followed by 32 cycles of denaturation at 94 °C for 30 s, annealing at a temperature shown in Table 1 for each fragment, respectively for 30 s, and extension at 72 °C for 1 min, as well as a final extension step at 72 °C for 7 min. Then, Sanger sequencing was performed in both directions on an Applied Biosystems 3500 Genetic Analyzer using a BigDye® Terminator v3.1 Cycle Sequencing Kit (Thermo Fisher Scientific, Waltham, MA, USA), according to the manufacturer’s instructions. The results were analyzed using the Sequencing Analysis Software system.

### Biopsy preparation

Approximately 1–2 mg of biopsies were obtained from sites of inflammed and non-inflamed regions from treatment-naïve patients with CD and from healthy controls during a colonoscopy. Next, the collected biopsies were suspended in 300 μl of RNALatter® reagent (Sigma) and frozen at − 80 °C until RNA isolation started.

### PBMCs isolation, culture, and treatment with the antibody

PBMCs were isolated from 9 ml samples of whole blood using LYMPHOSEP™ (MP Biomedicals LLC, OH, USA), according to the manufacturer’s instructions. The obtained pellet was suspended in 4 ml of a Lymphogrow medium (Cytogen-Polska Sp. z o.o., Zgierz, Poland) containing phytohemagglutinin (PHA) and recombinant IL-2 (4 ng, 100 U, BioLegend, San Diego, CA, USA). The suspension was then transferred to a 25-ml vessel for adherent culture. Cells were grown under standard conditions at 37 °C, 5% CO_2_ with shaking for 24 h. Non-adherent cells were washed with PBS and transferred to a 25-ml vessel for suspension culture with fresh Lymphogrow medium supplemented with IL2. After another 48 h, the cells were passaged and maintained in a culture using a standard RPMI-1630 medium supplemented with L-glutamine (2 mM), FBS (10%), penicillin (100 IU/ml), streptomycin (100 µg/ml), and with the addition of IL-2. Cell differentiation was measured by CD3, CD4, CD8, CD45, and HLA-DR by flow cytometry analysis. In the third passage, anti-TNF mAbs’ (Sigma) was added (10 µg/ml). In parallel, a control culture without the addition of the antibody was carried out. After 72 h of culture, cells were collected and suspended in 200 µl stayRNA solution (A&A Biotechnology, Gdansk, Poland) and frozen at − 80 °C for RNA isolation.

### *CASP9* gene expression studies

#### RNA isolation, cDNA synthesis, and quality control

Cells or mucosal tissue samples suspended in stayRNA™ (A&A BIOTECHNOLOGY, Gdansk, Poland) were homogenized with electric homogenizer and subjected to RNA isolation with TRIzol™ Reagent (Life Technologies, Carlsband, California), according to the manufacturer’s procedure. For all obtained RNA samples, a quantitative and qualitative evaluation was carried out using Agilent RNA 6000 Nano Kit and the Bioanalyzer 2.0 equipment (Agilent, Santa Clara, CA, USA). A 2 μg of total RNA with RIN ≥ 7 was converted to cDNA with an iScript Advanced Reverse Reaction kit (Bio-Rad) with the following conditions: 25 °C for 5 min—annealing step, 42 °C for 30 min—reverse transcription, and 95 °C for 1 min—inactivation.

#### Real-time quantitative PCR (RT-qPCR)

The mRNA level of the *CASP9* gene was measured by a real-time quantitative polymerase chain reaction on a BioRad CFX Connect 96-well Thermal Cycler (Bio-Rad Laboratories, Inc., Hercules, CA, USA) using the iTaq UniverSYBR Green assay (Bio-Rad Laboratories, Inc., Hercules, CA, USA), according to the manufacturer’s instructions. Specific primers: (1) forward 5′- GAGAATTGACCCTGGGGACAG-3′, reverse 5′- GCAGGACGCATCTCCAACG-3′ for amplification of 108 bp-length fragment of *CASP9* gene were designed by a Primer-BLAST tool. Primers for reference PPIA and RPLP0 genes were ordered as PrimePCRTM SYBR® Green Assay by Bio-Rad Laboratories, Inc. manufacturer. Results of qPCR reactions are presented as dCt = (dCtreference gene – dCtgene of interest). Every reaction was performed in duplicates.

### Bioinformatic and statistical analysis

The NGS reads generated in analysis were aligned to the hg19 reference genome using a Burrows–Wheeler Aligner (BWA. version 0.7.5) (Li and Durbin 2009). For PCR, duplicates marking Picard (version 2.1.0) were used. Local realignment around indels and a base quality score recalibration were carried out, followed by calling the variants by the Genome Analysis Toolkit (GATK version 3.5), in accordance with the GATK best practice procedure (McKenna et al. 2010). Next, single nucleotide variants were identified by means of using a HaplotypeCaller module and annotated by the VariantAnnotator module.

The comparison of interval data between responders and non-responders was conducted by nonparametric Mann–Whitney test, since the data did not follow the normal distribution pattern (Shapiro–Wilk test). The chi-square test was used for comparing nominal data, as well as to determine whether the association between the allele frequencies and the response to treatment was significant. Those analyses were performed using STATISTICA 13.3 software (StatSoft, Inc.) and PQStat 1.8.4 (PQStat Software, Poland) and all tests were considered significant at *p* < 0.05. After selecting variants that identified as statistically significant in the percentage of particular allele distribution between the group of responders and non-responders to anti-TNF treatment, in the next step, we used an odds ratio (OR) to demonstrate how many times more often a particular variant occurred in non-responder patients comparing to responder patients. OR is considered statistically significant when its 95% confidence interval (95% CI) does not contain 1. The analysis of genotypes distribution concordance with Hardy–Weinberg equilibrium and calculations of odds ratios (OR) with confidence intervals (CI) have been performed using the online calculator of Court-lab HW calculator (Court Lab—HW Calculator, (scribd.com) accessed on date 25.07.2023).

For the RT-qPCR data, in the case of lymphocytes, the Mann–Whitney non-parametric test was used to compare median value of the three groups—controls, responders and non-responding patients in reference to conditions with and without anti-TNF infliximab antibody. Comparison between tissue samples from controls, responders (inflammed and non-inflammed) and non-responders (inflammed and non-inflammed tissue) were conducted with Kruskal–Wallis one-way analysis of variance. *P*-values < 0.05 were considered as statistically significant. All analyses were performed using R software 3.6.1 and RStudio.

## Results

Based on NGS results of *CASP9* gene in a studied group of 96 CD patients, we observed in total of 55 variants in exons 4–9. For the first amplicon containing 5′UTR and exons 1–3, the obtained NGS data did not meet quality filtering as sequencing depth > 30 reads; therefore, they were discarded and replicated using Sanger sequencing as shown below. Among identified variants, one was located in exon (rs1052576, c.662A > G), 51 in introns (one of them in splice region, rs2020902, c.453 + 8 T > C) and three in 3′UTR (Table S4). Compering alleles distribution in responders and non-response to anti-TNF treatment, 29 variants revealed statistical significance differences based on chi-square *p*-value < 0.05. However, after multiple corrections, the statistical significance was not rich (Table S4).

The standard Sanger sequencing method was used to verify the NGS results as well as to complete the promoter region and exons 1, 2, and 3 as well as extension the study group to 196 CD patients. Obtained data of Sanger sequencing showed significant association of two variants rs1052571 and rs4645981with response to anti-TNF as detailed in Table 2 (*p* < 0.05). The results for the third variant rs4645978 are near statistical significance (*p* = 0.07).

**Table 2:** *CASP9* variants associated with response to anti-TNF agents

No	*CASP9* variant		Responders, *n* = 163					HWE		Non-responders, *n* = 33					HWE		Responders vs. non-responders	
			Genotypes			Alleles		χ^2^	*p*	Genotypes			Alleles		χ^2^	*p*	χ^2^	*p*
			11	12	22	1	2			11	12	22	1	2				
1	**rs1052571** c.83C > T	*n*	46	66	51	158	168	5.85	0.0156	16	7	10	39	27	10.40	0.0013	6.31	0.0426
		%	28.2	40.5	31.3	48.5	51.5			48.5	21.2	30.3	59.1	40.9				
2	**rs4645978** c.-239 + 652G > T	*n*	30	69	64	129	197	2.15	0.1425	8	19	6	35	31	0.80	0.3709	5.32	0.0700
		%	18.4	42.3	39.3	39.6	60.4			24.2	57.6	18.2	53.0	47.0				
3	**rs4645981** c.-239 + 1203C > G	*n*	35	61	67	131	195	8.00	0.0047	3	21	9	27	39	3.30	0.0693	8.03	0.0180
		%	21.5	37.4	41.1	40.2	59.2			9.1	63.6	27.3	40.9	59.1				

**Fig. 2:**
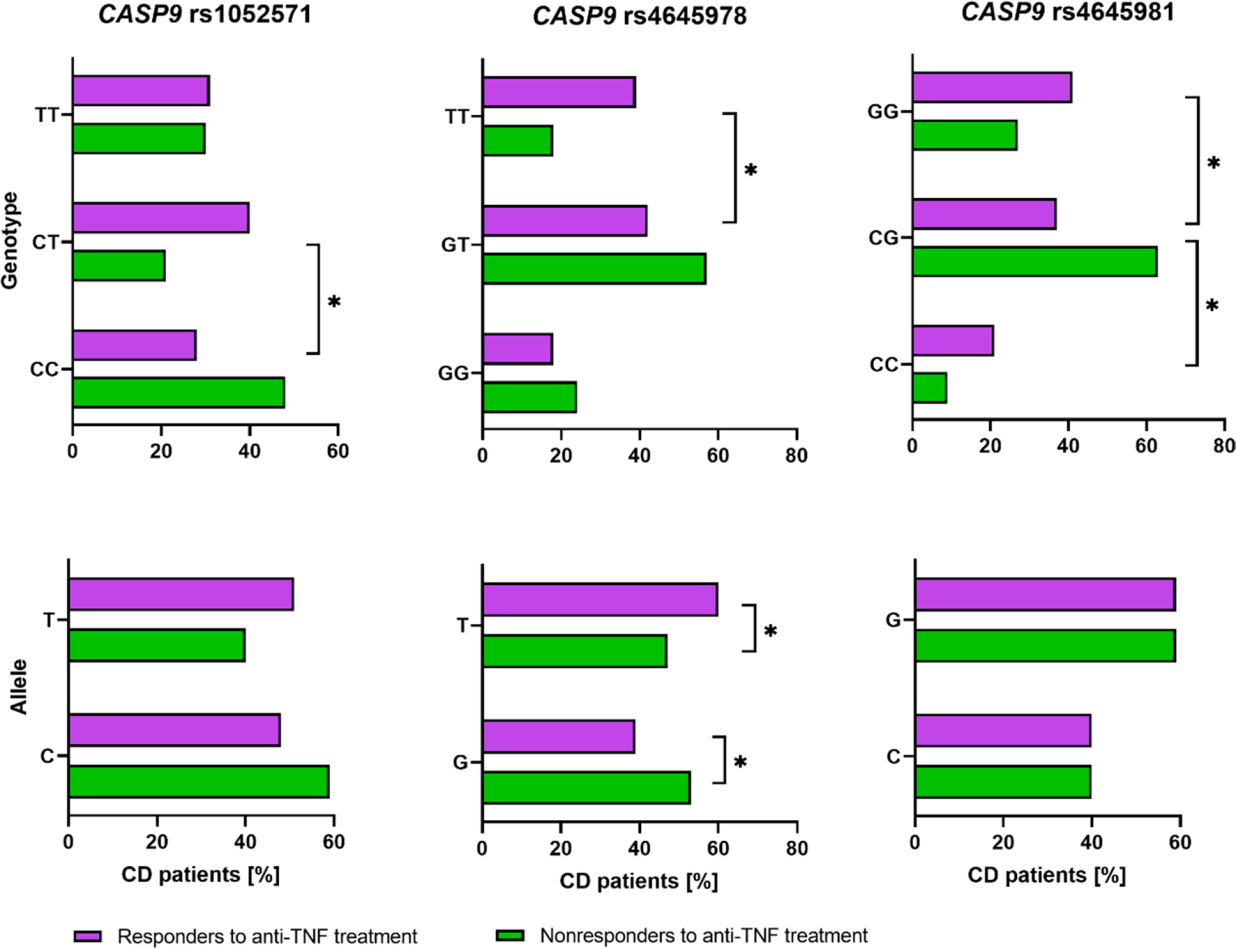
Distribution of genotypes and alleles for significantly associated variants of the *CASP9* gene. *, *p* < 0.05

We observed that rs1052571 was non consistent with Hardy–Weinberg equilibrium (HWE) in both studied groups, whereas rs4645981 was deviations only in the responders patients’ group (*p* < 0.05). Detailed comparison of allelic and genotypic distributions between responders and non-responders to anti-TNF treatment is shown in Table 3 and Fig. 2.

**Table 3:** Comparisons of allelic and genotypic frequencies between groups of responders and non-responders to anti-TNF treatment

*CASP9* variant (major > minor allele)	Allele frequency difference		Heterozygous		Homozygous		Allele positivity	
	[1] vs. [2]	[2] vs. [1]	[11] vs. [12]	[22] vs. [12]	[11] vs. [22]	[22] vs. [11]	11 vs. [22 + 12] Non-response risk allele 2	[11 + 12] vs. 22 Non-response risk allele 1
rs1052571 c.83C > T p.Ala28Val	OR = 1.54 95%CI[0.90–2.63]	OR = 0.65 95% CI [0.38–1.11]	OR = 3.28 95%CI[1.25–8.61] *p* = 0.0125	OR = 1.85 95%CI[0.66–5.19] *p* = 0.2384	OR = 1.77 95%CI[0.73–4.30]	OR = 0.56 95%CI[0.23–1.37]	OR = 2.40 95%CI[1.12–5.14] *p* = 0.0224	OR = 1.05 95%CI[0.47–2.36] *p* = 0.9203
	*p* = 0.1153				*p* = 0.2017			
rs4645978 c.-239 + 652G > T	OR = 1.72 95%CI[1.01–2.94]	OR = 0.58 95%CI[0.34–0.99]	OR = 0.10 95%CI[0.38–2.46] *p* = 1.0	OR = 0.34 95%CI[0.13–0.91] *p* = 0.0259	OR = 2.84 95%CI[0.91–8.93]	OR = 0.35 95%CI[0.11–1.10]	OR = 1.42 95%CI[0.58–3.45] *p* = 0.4386	OR = 3.00 95%CI[1.17–7.68] *p* = 0.0177
	*p* = 0.0431				*p* = 0.0636			
rs4645981 c.-239 + 1203C > G	OR = 1.03 95%CI[0.60–1.77]	OR = 0.97 95%CI[0.57–1.66]	OR = 0.25 95%CI[0.07–0.90] *p* = 0.0241	OR = 0.39 95%CI[0.17–0.92] *p* = 0.0275	OR = 0.64 95%CI[0.16–251]	OR = 1.57 95%CI[0.4–6.16]	OR = 0.37 95%CI[0.11–1.27] *p* = 0.101	OR = 1.86 95%CI[0.81–4.26] *p* = 0.1371
	*p* = 0.9203				*p* = 0.3841			

^a^, results significant *p* < 0.05; 1, the first allele (major); 2, the second allele (minor); 11—the first type of homozygous genotype; 22—the second type of homozygous genotype; 12, genotype heterozygote; [11 + 12] versus [22], dominant model with risk allele 1; [11] versus [12 + 22], recessive model with risk allele 2; [GG + GA] versus [AA], dominant model, risk allele: G; [GG] versus [GA + AA], recessive model, risk allele A; 95% CI confirming the statistically significant odds ratio (OR)

**Fig. 3:**
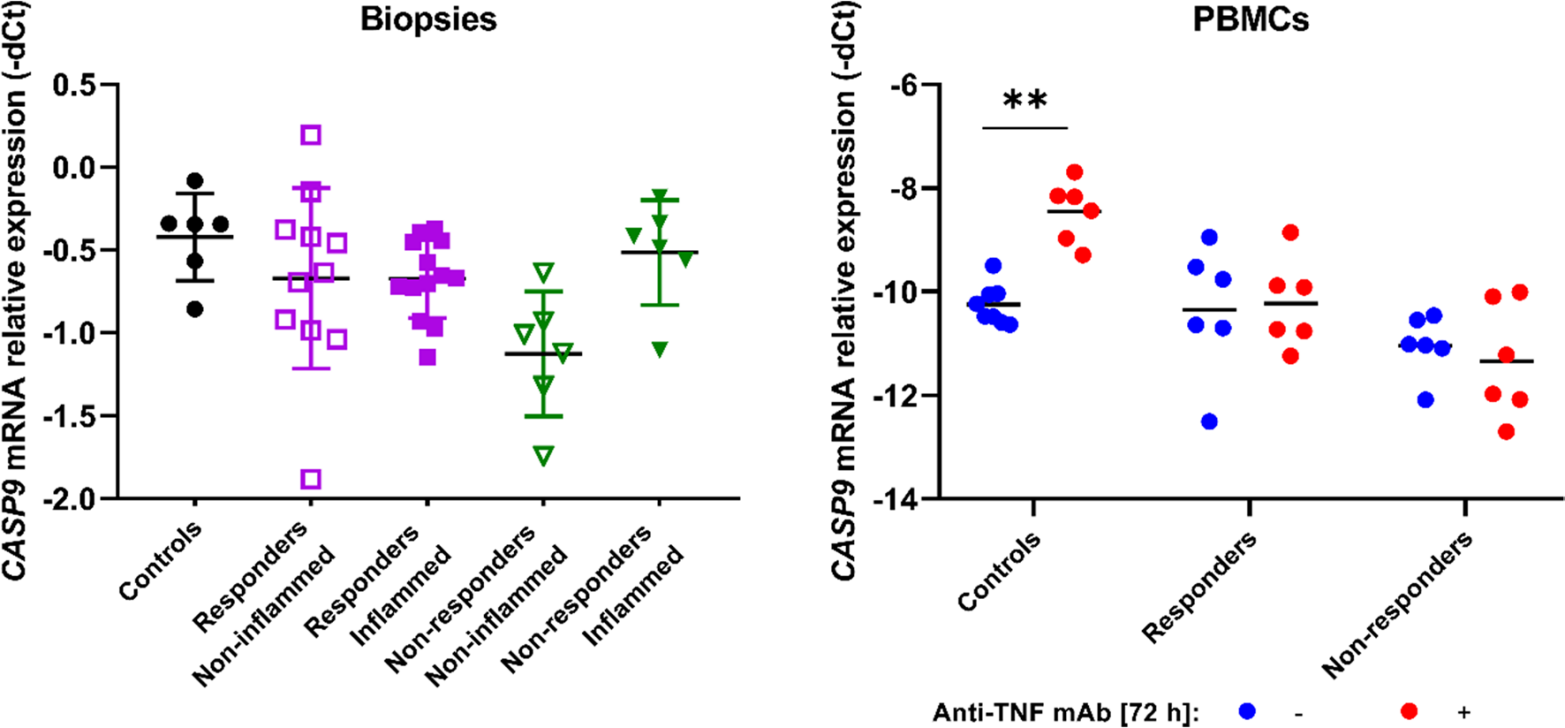
Relative expression of the *CASP9* gene in CD patients’ colon biopsies and in PBMCs culture treated with anti-TNF mAbs

In the last part of experiments, the *CASP9* gene mRNA expression was estimated in CD patients colon biopsies and in PBMCs culture treated with anti-TNF. Based on results, no statistically significant differences in the expression of *CASP9* gene were found in any of the subgroups studied; however, in the case of cell cultures, there is a tendency for reduced expression after incubation with anti-TNF in the group of patients, in contrast to the control group, in which expression increases (Fig. 3, Table S5, Table S6).

## Discussion

Impaired adaptive immunity of cells of the intestinal immune system is crucial for the development and maintenance of inflammation in IBD. Lymphocyte apoptosis plays an important role in the breakdown of inflammation. Cell death is initiated by activation of ligands, such as TNF-α or FAS that bind to appropriate death receptors—in the external pathway or by the release of mitochondrial components, such as cytochrome c—in the internal pathway. Disruption of the apoptosis process can lead to many pathologies, such as cancer or autoimmune diseases. A characteristic feature of CD is the infiltration of T lymphocytes in the intestinal mucosa. In patients with IBD, an increased level of anti-apoptotic mediators in the intestinal mucosa and the abnormal response of T lymphocytes to pro-apoptotic signals is observed (Sturm et al. 2002). Interestingly, one of the modes of action of anti-TNF drugs is the ability to induce lymphocyte apoptosis (Makrygiannakis and Catrina 2012); however, the exact mechanism of the influence of these drugs on the phenomenon of apoptosis remains elusive.

The studies conducted so far showed that in the T lymphocytes of the lamina propria of patients with CD, increased activation of the anti-apoptotic protein Bcl-2, which results in the inhibition of the internal apoptotic pathway, is observed (Lindsay et al. 2011; Eder et al. 2013b). CASP-9 is one of the main factors of the internal apoptosis pathway. Cytochrome c, together with CASP-9 and Apaf-1, creates the so-called apoptosome, which activates caspase-3, the main enforcer of apoptosis. Hlavaty et al. observed that patients with infliximab-resistant luminal and fistula form of the disease, carried the rs4645983 genotype CC and CT of the *CASP9* with higher frequency (*p* = 0.04; *OR* = 1.50; 95% CI [1.34–1.68]) (Hlavaty et al. 2005). In our study, we did not confirm this association. However, we identified two other variants related to response to anti-TNF: rs1052571 and rs4645981. Results for the third variant, rs4645978 are near statistical significance (*p* = 0.07). Interestingly, the variant rs1052571 described by us is located at position c.83 of the *CASP9* gene, i.e., 10 nucleotides prior to exon 1 than rs4645983 described by Hlavaty et al. and leads to the substitution of the amino acid alanine to valine at position 28 (p.Ala28Val). We found that homozygote TT occurred with lower frequency in responders compared to non-responders (28.2% vs. 48.5%) and may promote response to anti-TNF therapy (*OR* = 2.40, 95% CI [1.12–5.14], *p* = 0.0224, Table 3). Therefore, homozygote CC is associated with non-response (*OR* = 3.28, 95% CI [1.25–8.61], *p* = 0.0125). Guo and colleagues studied this variant in a group of 555 patients with CD and 651 patients with UC, who attempted to assess the correlation between *CASP9* gene haplotypes and susceptibility to IBD. They showed that rs1052571 in the *CASP9* gene was associated with severe UC (*p* = 0.0034, *OR* = 1.957, 95% CI [1.240–3.088]). These findings suggest that the *CASP9* gene may be another susceptibility gene for severe IBD (Guo et al. 2011). It cannot be ruled out that the polymorphism of this gene is also related to non-response to drugs, which we showed in our research. In addition to this relationship, we also observed that two promoter variants are associated with response to anti-TNF treatment. T allele of rs4645978 (c.-239 + 652G > T) was detected much more often in responders (60.4% vs. 47% respectively), and the GG and GT genotype was three times more common in non-responders (*OR* = 3.00, 95% CI [1.17–7.68],* p* = 0.0177), and the TT genotype conditioned a better response to drugs (*OR* = 0.34, 95% CI [0.13–0.91], *p* = 0.0259). The second variant of the promoter region shown by us in this study is rs4645981 (c.-239 + 1203C > G), where the presence of heterozygote CG was associated with non-response to anti-TNF treatment in patients with CD. Although we observed a statistical dependence in the distribution of genotypes, the share of C and G alleles is similar in both study groups. Therefore, it is difficult to postulate the significance of this variant in response to anti-TNF drugs, in contrast to the two variants described above. The location of the rs4645978 variant in the gene promoter may affect the expression level of the *CASP9* gene and the protein level. Therefore, the continuation of our observations was the evaluation of CASP-9 protein expression in colon tissues from patients with CD and healthy individuals. We found no differences in *CASP9* mRNA expression between these groups. Our observations suggest no change in the induction of the internal apoptosis pathway in active CD. However, it cannot be ruled out that the polymorphisms described by us have a negative impact on the function of the CASP-9 protein and its expression. The limitation of our observation is the small number of examined tissues.

It might not be excluded that the variants described by us have a negative impact on the function and expression of the CASP-9 protein. The limitation of our observation is a small number of examined tissues. However, we observed that in patients with active Crohn’s disease, the internal apoptosis pathway is not significantly enhanced, especially in the group of non-responders (Fig. 3). Our observations are consistent with the results obtained by Neubauer et al. They assessed CASP-9 concentration in peripheral blood lymphocytes of patients with IBD by immunoenzymatic method and showed significantly lower concentrations of CASP-9 in patients with active IBD compared to the control group (Neubauer et al. 2018). A follow-up study of our experiment was to determine CASP-9 mRNA expression in cell cultures of peripheral blood lymphocytes that were treated with IFX. We showed that CASP-9 expression is reduced under the influence of IFX in non-responding patients. No correlation was observed in healthy subjects and in responders. Our observations are consistent with the observations of Eder et al., who, using immunohistochemical methods, showed that in patients with active CD responding to infliximab treatment, there is a significant increase in the expression of active caspase-3 in the mononuclear cells of the lamina propria, which correlated with an increase in the pro-apoptotic Bax/Bcl-2 pathway. These differences were not observed in non-responding patients (Eder et al. 2013a). Similar results were obtained by other researchers. Ten Hove et al. demonstrated in an in vitro model that IFX induces apoptosis and increases the Bax/Bcl-2 ratio of CD3/CD28-stimulated Jurkat T cells (Ten Hove et al. 2002).

Although many researchers suggest that anti-TNF drugs do not directly induce apoptosis of inflammatory cells, it seems that damage to the internal pathway may be critical in resistance to biological treatment. Undoubtedly, the limitation of our study is the small number of patients. However, there is a clear tendency that patients who did not respond to the treatment did not show an increase in *CASP9* gene expression when compared to the control group and the group of patients responding to the drug.

In summary, our results suggest that response to anti-TNF therapy in CD patients could be an effect of variants of *CASP9* gene as a key effector of the internal pathway of apoptosis. However, more detailed research is needed in this area.

### Supplementary Information

Below is the link to the electronic supplementary material.

Supplementary file1 (DOCX 50 KB)

## Data Availability

All primary data are available from the corresponding author upon the reasonable request.
