# Supplementary material for: Variants of the CASP9 gene as candidate markers for primary response to anti-TNF therapy in Crohn’s disease patients
Source: J Appl Genet. 2023 Sep 2;64(4):759–68. doi: 10.1007/s13353-023-00783-7 (PMC10632275; doi:10.1007/s13353-023-00783-7)
Supplement: Supplementary file 1 — Supplementary file1 (DOCX 50 KB) [file 13353_2023_783_MOESM1_ESM.docx]

**SUPPLEMENTARY INFORMATION**

**Table S1** The characteristics of patients in *CASP9* gene sequencing studies

| **Parameter** | **All patients,**  **n = 196** | **Responders,**  **n = 163** | **Non-responders,**  **n = 33** | ***p*-value** |
| --- | --- | --- | --- | --- |
| **Gender F/M, n (%)** | 77 (39.29%)/  119 (60.71%) | 62 (38.04%)/  101 (61.96%) | 15 (45.45%)/  18 (54.55%) | 0.43 |
| **Age, years, mean ± SD** | 30.65 **±** 10.58  (17-64) | 30.03 ± 9.77  (17-64) | 33.73 ± 13.64  (19-64) | 0.25 |
| **Smoker** | 9 (4.59%) | 9 (5.52%) | 0 (0.00%) | 0.17 |
| **Previous** **operations** | 67 (34.18%) | 49 (30.06%) | 18 (54.55%) | 0.007 |
| **CRP^a^, mg/l, median IQR** | 18.6  (6.30-40.70) | 14  (5.93-35.23) | 37  (18.95-60.40) | 0.001 |
| **CRP^b^, mg/l, median IQR** | 3.95  (1.2-13.18) | 2.9  (1-8.40) | 26  (10.10-54.7) | <0.0001 |
| **CDAI^a^, pts, median IQR** | 323  (300-344.3) | 320  (290-340) | 340  (320-350) | 0.0008 |
| **CDAI^b^, pts, median IQR** | 190  (150-220) | 180  (150-200) | 300  (285-305) | <0.0001 |
| **SES CD^a^, mean ± SD** | 9.89 ± 3.72 | 9.94 ± 3.78 | 9.56 ± 3.37 | 0.7102 |
| **SES CD^b^, mean ± SD** | 5.84 ± 3.32 | 5.43 ± 3.09 | 8.2 ± 2.34 | 0.0013 |
| **SEAS CD^a^, mean ± SD** | 8.47 ± 3.32 | 8.43 ± 3.54 | 8.59 ± 2.40 | 0.84 |
| **SEAS CD^b^, mean ± SD** | 5.85 ± 2.78 | 5.41 ± 2.74 | 7.87 ± 2.07 | 0.0097 |
| **Disease duration, months, mean ± SD (min.-max.)** | 60.67 ± 60.67  (3-348) | 65.77 ± 74.76  (3-620) | 69.88 ± 65.49  (3-234) | 0.90 |
| **Intestinal location^c^** |  |  |  |  |
| **Ileal (L1)** | 51 (26.02%) | 40 (24.54%) | 11 (33.33%) | 0.29 |
| **Colonic (L2)** | 68 (34.69%) | 58 (35.59%) | 10 (30.30%) | 0.56 |
| **Ileocolonic (L3)** | 77 (39.29%) | 65 (39.88%) | 12 (36.36%) | 0.71 |
| **Behavior** |  |  |  |  |
| **Nonstricturing, nonpenetrating (B1)** | 113 (57.65%) | 94 (57.67%) | 19 (57.58%) | 0.99 |
| **Stricturing (B2)** | 33 (16.84%) | 28 (17.18%) | 5 (15.15%) | 0.78 |
| **Penetrating (B3)** | 1 (0.51%) | 1 (0.61%) | 0 (0.00%) | 0.65 |
| **Perianal disease modifier (P)** | 51 (26.02%) | 42 (25.77%) | 9 (27.27%) | 0.86 |
| **Medication** |  |  |  |  |
| **Mesalamine** | 193 (98.47%) | 161 (98.77%) | 32 (96.97%) | 0.44 |
| **Corticosteroids** | 59 (30.10%) | 49 (30.06%) | 10 (30.30%) | 0.98 |
| **Azathioprine** | 111 (56.63%) | 96 (58.89%) | 15 (45.45%) | 0.16 |
| **Inxliximab** | 139(70.92%) | 115 (70.55%) | 24 (72.73%) | 0.80 |
| **Adalimumab** | 57 (29.08%) | 48 (29.45%) | 9 (27.27%) | 0.80 |

IQR, interquartile range**; ^a^**, before anti-TNF treatment; **^b^**, after 3 months anti-TNF treatment; **^c^**, Disease locations were classified according to the Montreal Classification (Satsangi et al. 2006).

**Table S2** Characteristics of the patients group in the mucosa studies

| **Parameter** | **Total patients**  **n = 21** | **Responders**  **n = 14** | **Non-responders**  **n = 7** | ***p*-value** |
| --- | --- | --- | --- | --- |
| **Gender, (F/M), n (%)** | 10 (47.61)/11(52.39) | 7 (50.00)/7 (50.00) | 3 (42.86)/4 (57.14) | 0.7574 |
| **Age, (years) mean ± SD** | 33.81 ± 12.51 | 30.64 ± 8.21 | 40.14 ± 17.47 | 0.2858 |
| **Smoker, n (%)** | 1 (4.76) | 1 (7.14) | 0 (0.00) | 0.4687 |
| **Previous surgeries, n (%)** | 8 (38.09) | 2 (14.28) | 6 (85.71) | 0.0015 |
| **Disease duration, months, mean ± SD** | 59.62 ± 42.42 | 53.29 ± 35.51 | 72.29 ± 54.63 | 0.5190 |
| **Intestinal location,* n (%)** | | | | |
| **Colonic (L2)** | 6 (28.57) | 3 (21.43) | 3 (42.86) | 0.3055 |
| **Ileal (L1)** | 4 (19.05) | 3 (21.43) | 1 (14.28) | 0.6944 |
| **Ileocolonic (L3)** | 11 (52.38) | 8 (57.14) | 3 (42.86) | 0.5366 |
| **Behavior, n (%)** | | | | |
| **Nonstricturing nonpenetrating (B1)** | 21 (100.00) | 14 (100.00) | 7 (100.00) | >0.9999 |
| **Stricturing (B2)** | 0 (0.00) | 0 (0.00) | 0 (0.00) | >0.9999 |
| **Penetrating (B3)** | 0 (0.00) | 0 (0.00) | 0 (0.00) | >0.9999 |
| **Medication, n (%)** | | | | |
| **Mesalamine** | 21 (100.00) | 14 (100.00) | 7 (100.00) | >0.9999 |
| **Corticosteroids** | 8 (38.90) | 4 (28.57) | 4 (57.14) | 0.2037 |
| **Azathioprine** | 13 (61.90) | 9 (63.28) | 4 (57.14) | 0.7507 |
| **Adalimumab** | 4 (19.05) | 3 (21.43) | 1 (14.28) | 0.6944 |
| **Infliximab** | 17 (80.95) | 11 (78.57) | 6 (85.71) | 0.6944 |

*Disease locations were classified according to the Montreal Classification (Satsangi et al. 2006).

**Table S3** Characteristics of patients group in PBMC culture studies

| **Parameter** | **Total patients**  **n = 14** | **Responders**  **n = 7** | **Non-responders**  **n = 7** | ***p*-value** |
| --- | --- | --- | --- | --- |
| **Gender, F/M, n (%)** | 4 (28.57)/10 (71.43) | 2 (28.57)/5 (71.43) | 2 (28.57)/5 (71.43) | >0.9999 |
| **Age, (years) mean ± SD** | 29.86 ± 7.29 | 28.71 ± 6.08 | 31 ± 8.68 | 0.7372 |
| **Smoker, n (%)** | 0 (0.00) | 0 (0.00) | 0 (0.00) | >0.9999 |
| **Previous operations, n (%)** | 6 (42.86) | 2 (28.57) | 4 (57.14) | 0.2801 |
| **Disease duration, months, mean ± SD** | 61.71 ± 50.44 | 65.14 ± 47.93 | 58.29 ± 56.47 | 0.5233 |
| **Intestinal location,* n (%)** | | | | |
| **Colonic (L2)** | 4 (28.57) | 2 (28.57) | 2 (28.57) | >0.9999 |
| **Ileal (L1)** | 2 (14.29) | 2 (28.57) | 0 (0.00) | 0.1266 |
| **Ileocolonic (L3)** | 7 (50.00) | 3 (42.86) | 5 (71.43) | 0.2801 |
| **Behavior, n (%)** | | | | |
| **Non-stricturing, non-penetrating (B1)** | 10 (71.43) | 6 (85.71) | 4 (57.14) | 0.2367 |
| **Stricturing (B2)** | 2 (14.29) | 1 (14.28) | 1 (14.28) | >0.9999 |
| **Penetrating (B3)** | 2 (14.29) | 0 | 2 (28.57) | 0.1266 |
| **Medication, n (%)** | | | | |
| **Mesalamine** | 14 (100.00) | 7 (100.00) | 7 (100.00) | >0.9999 |
| **Corticosteroids** | 4 (28.57) | 1 (14.28) | 3 (42.86) | 0.2367 |
| **Azathioprine** | 8 (57.14) | 3 (42.86) | 5 (71.43) | 0.2801 |
| **Adalimumab** | 6 (42.86) | 3 (42.86) | 3 (42.86) | >0.9999 |
| **Infliximab** | 7 (50.00) | 4 (57.14) | 3 (42.86) | 0.5930 |

*Disease locations were classified according to the Montreal Classification (Satsangi et al. 2006).

**Table S4** NGS results of *CASP9* gene

| **Rs number** | **DNA variant** | **Location** | **Resp.**  **MAF [%]** | **Non- resp. MAF [%]** | **ꭓ^2^** | ***p*-value** | ***p* adj.** |
| --- | --- | --- | --- | --- | --- | --- | --- |
| rs884363 | c.*511G>T | 3’ UTR | 36.05 | 70.00 | 5.56 | 0.0184 | 0.3373 |
| rs4646103 | c.*452A>T | 3’ UTR | 25.00 | 30.00 | 0.03 | 0.8595 | 1.0000 |
| rs4646100 | c.*238G>A | 3’ UTR | 43.60 | 20.00 | 5.23 | 0.0222 | 0.2439 |
| rs2020900 | c.1049-80A>T | Intron | 36.05 | 70.00 | 5.56 | 0.0184 | 0.2530 |
| rs2020895 | c.1049-111A>G | Intron | 38.37 | 70.00 | 4.89 | 0.0271 | 0.0930 |
| rs2020898 | c.1049-122T>C | Intron | 36.05 | 70.00 | 5.95 | 0.0147 | 0.4048 |
| rs4646096 | c.1049-262T>C | Intron | 25.00 | 30.00 | 0.03 | 0.8595 | 1.0000 |
| rs10803387 | c.1049-555G>T | Intron | 43.60 | 20.00 | 5.23 | 0.0222 | 0.2032 |
| rs9700738 | c.1048+604C>G | Intron | 25.00 | 30.00 | 0.03 | 0.8595 | 1.0000 |
| rs4646093 | c.1048+368G>T | Intron | 43.02 | 20.00 | 5.02 | 0.0250 | 0.1146 |
| rs4233532 | c.1048+183G>A | Intron | 39.53 | 70.00 | 4.24 | 0.0396 | 0.0807 |
| rs4646092 | c.1048+95G>A | Intron | 25.00 | 30.00 | 0.04 | 0.8375 | 1.0000 |
| rs4646091 | c.869-96A>G | Intron | 25.00 | 30.00 | 0.03 | 0.8595 | 1.0000 |
| rs4646090 | c.869-126G>A | Intron | 43.60 | 20.00 | 5.07 | 0.0244 | 0.1218 |
| rs4646087 | c.869-457T>C | Intron | 25.00 | 30.00 | 0.03 | 0.8595 | 1.0000 |
| rs4661636 | c.869-1114G>A | Intron | 26.16 | 55.00 | 4.93 | 0.0264 | 0.0967 |
| rs6429747 | c.869-1942G>A | Intron | 43.02 | 20.00 | 5.02 | 0.0250 | 0.1058 |
| rs35328037 | c.869-2205T>A | Intron | 25.00 | 30.00 | 0.03 | 0.8595 | 0.9848 |
| rs34094056 | c.869-2207C>A | Intron | 25.00 | 30.00 | 0.03 | 0.8595 | 0.9647 |
| rs72643683 | c.869-2958G>T | Intron | 25.00 | 30.00 | 0.04 | 0.8375 | 1.0000 |
| rs6685648 | c.869-3248A>G | Intron | 43.60 | 20.00 | 5.23 | 0.0222 | 0.1742 |
| rs4646085 | c.869-3464T>A | Intron | 25.00 | 30.00 | 0.03 | 0.8595 | 0.9455 |
| rs4646083 | c.869-3617C>T | Intron | 25.00 | 30.00 | 0.03 | 0.8595 | 0.9269 |
| rs951225 | c.869-4345C>T | Intron | 25.58 | 30.00 | 0.01 | 0.9067 | 0.9235 |
| rs4646080 | c.868+4479T>A | Intron | 24.42 | 30.00 | 0.04 | 0.8341 | 1.0000 |
| rs11589076 | c.868+4079G>A | Intron | 24.42 | 30.00 | 0.06 | 0.8121 | 1.0000 |
| rs4646077 | c.868+3970G>A | Intron | 25.00 | 30.00 | 0.03 | 0.8595 | 0.9091 |
| rs577634487 | c.868+3816C>T | Intron | 3.49 | 5.00 | 0.05 | 0.817 | 1.0000 |
| rs4646075 | c.868+3645A>G | Intron | 22.67 | 25.00 | 0.01 | 0.9467 | 0.9467 |
| rs4646072 | c.868+3395C>T | Intron | 24.42 | 25.00 | 0.05 | 0.8262 | 1.0000 |
| rs4646068 | c.868+2402A>G | Intron | 43.60 | 20.00 | 5.23 | 0.0222 | 1.0000 |
| rs4233533 | c.868+1919C>T | Intron | 43.02 | 20.00 | 5.02 | 0.025 | 0.0982 |
| rs4646066 | c.868+1880A>T | Intron | 38.95 | 70.00 | 4.29 | 0.0383 | 0.0958 |
| rs4661637 | c.868+1649G>C | Intron | 37.21 | 70.00 | 4.84 | 0.0278 | 0.0901 |
| rs4646064 | c.868+1303G>A | Intron | 5.81 | 5.00 | 0.07 | 0.794 | 1.0000 |
| rs4646063 | c.868+1293C>T | Intron | 25.58 | 25.00 | 0.11 | 0.7381 | 1.0000 |
| rs4646061 | c.868+1190G>A | Intron | 43.60 | 20.00 | 5.2 | 0.0222 | 0.1355 |
| rs4646059 | c.868+1047T>G | Intron | 38.95 | 70.00 | 4.29 | 0.0383 | 0.0917 |
| rs4646058 | c.868+836C>A | Intron | 38.37 | 70.00 | 4.5 | 0.0334 | 0.0966 |
| rs4646049 | c.721-207A>G | Intron | 10.47 | 10.00 | 0.05 | 0.8208 | 1.0000 |
| rs4646047 | c.721-530A>G | Intron | 39.53 | 70.00 | 4.06 | 0.0436 | 0.0861 |
| rs4646045 | c.720+493T>C | Intron | 38.95 | 70.00 | 4.29 | 0.0383 | 0.0878 |
| rs4646044 | c.720+418G>A | Intron | 37.79 | 70.00 | 4.59 | 0.0322 | 0.0984 |
| rs4646043 | :c.720+375A>C | Intron | 5.81 | 10.00 | 0.33 | 0.5668 | 0.9742 |
| rs1800616 | c.720+256C>T | Intron | 25.00 | 30.00 | 0.03 | 0.8595 | 0.8919 |
| rs1800615 | c.720+204G>A | Intron | 43.60 | 20.00 | 5.23 | 0.0222 | 0.1219 |
| rs1800614 | c.720+102C>A | Intron | 38.95 | 70.00 | 4.29 | 0.0383 | 0.0843 |
| rs1052576 | c.662A>T>G | Exon | 38.37 | 70.00 | 4.53 | 0.0334 | 0.0918 |
| rs4646040 | c.631-333G>A | Intron | 36.05 | 75.00 | 7.56 | 0.006 | 0.3291 |
| rs4646036 | c.454-338G>A | Intron | 40.12 | 70.00 | 3.84 | 0.05 | 0.0948 |
| rs4646034 | c.453+208T>C | Intron | 38.95 | 70.00 | 4.29 | 0.0383 | 0.0811 |
| rs2020903 | c.453+71C>T | Non coding transcript exon | 38.95 | 65.00 | 2.67 | 0.1011 | 0.1854 |
| rs2020902 | c.453+8T>C | Splice region | 10.47 | 10.00 | 0.05 | 0.8208 | 1.0000 |
| rs4646032 | c.419-89C>T | Intron | 36.05 | 70.00 | 4.53 | 0.0334 | 0.0874 |
| rs4646031 | c.419-246T>C | Intron | 25.00 | 35.00 | 0.39 | 0.5319 | 0.9437 |

*p*-adj. - *p*-value adjusted

**Table S5** Mucosal expression of *CASP9* mRNA in CD patients and controls

| Groups | Number | ∆Ct  (Median) | Q1-Q3 | *p*-value |
| --- | --- | --- | --- | --- |
| Responders non-inflammed mucosa (RN) | 11 | -0.64 | -0.95; -0.40 | 0.3579 (RN *vs.* C)  0.3956 (RN *vs.* NN)  0.4261 (RN *vs.* NI)  0.7531 (RN *vs.* RI) |
| Responders inflammed mucosa (RI) | 13 | -0.69 | -0.94; -0.54 | 0.2965 (RI *vs.* C)  0.3463 (RI *vs.* NN)  0.3090 (RI *vs.* NI) |
| Non-responders non-inflammed mucosa (NN) | 6 | -1.01 | -1.38; -0.82 | 0.2439 (NN *vs.* C)  0.2367 (NN *vs.* NI) |
| Non-responders inflammed mucosa (NI) | 6 | -0.45 | -0.54; -0.36 | 0.6900 (NI *vs.* C) |
| Controls | 6 | -0.4562 | -0.64;-0.28 | - |

Q1, first quartile; Q3; third quartile

**Table S6** PBMC expression of *CASP9* mRNA in CD patients and controls

| Groups | Number | ∆Ct  (Median) | Q1-Q3 | *p*-value |
| --- | --- | --- | --- | --- |
| Controls (-) | 8 | -10.36 | -10.51; -10.05 | 0.0007 |
| Controls (+) | 6 | -8.303 | -8.836; -8.155 |  |
| Responders (-) | 6 | -10.205 | -10.690; -9.585 | 0.6991 |
| Responders (+) | 6 | -10.322 | -10.756; -9.889 |  |
| Nonresponders (-) | 6 | -10.83 | -11.07; -10.57 | 0.3939 |
| Nonresponders (+) | 6 | -12.02 | -12.18; -10.57 |  |

Q1, first quartile; Q3, third quartile
